# Changes in Ghrelin-Related Factors in Gastroesophageal Reflux Disease in Rats

**DOI:** 10.1155/2013/504816

**Published:** 2013-04-04

**Authors:** Miwa Nahata, Yayoi Saegusa, Yumi Harada, Naoko Tsuchiya, Tomohisa Hattori, Hiroshi Takeda

**Affiliations:** ^1^Tsumura & Co., Tsumura Research Laboratories, 3586 Yoshiwara, Ami-machi, Inashiki-gun, Ibaraki 300-1192, Japan; ^2^Department of Gastroenterology and Hematology, Hokkaido University Graduate School of Medicine, N15 W7 Kita-ku, Sapporo, Hokkaido 060-8638, Japan; ^3^Department of Pathophysiology and Therapeutics, Division of Pharmasciences, Faculty of Pharmaceutical Sciences, Hokkaido University, N12 W6 Kita-ku, Sapporo, Hokkaido 060-0812, Japan

## Abstract

To examine gastrointestinal hormone profiles and functional changes in gastroesophageal reflux disease (GERD), blood levels of the orexigenic hormone ghrelin were measured in rats with experimentally induced GERD. During the experiment, plasma acyl ghrelin levels in GERD rats were higher than those in sham-operated rats, although food intake was reduced in GERD rats. Although plasma levels of the appetite-suppressing hormone leptin were significantly decreased in GERD rats, no changes were observed in cholecystokinin levels. Repeated administration of rat ghrelin to GERD rats had no effect on the reduction in body weight or food intake. Therefore, these results suggest that aberrantly increased secretion of peripheral ghrelin and decreased ghrelin responsiveness may occur in GERD rats. Neuropeptide Y and agouti-related peptide mRNA expression in the hypothalamus of GERD rats was significantly increased, whereas proopiomelanocortin mRNA expression was significantly decreased compared to that in sham-operated rats. However, melanin-concentrating hormone (MCH) and prepro-orexin mRNA expression in the hypothalamus of GERD rats was similar to that in sham-operated rats. These results suggest that although GERD rats have higher plasma ghrelin levels, ghrelin signaling in GERD rats may be suppressed due to reduced MCH and/or orexin synthesis in the hypothalamus.

## 1. Introduction

Gastroesophageal reflux disease (GERD) is caused when gastric acid flows back into the esophagus, resulting in erosion of the esophageal mucosal epithelium. Gastric secretion inhibitors, such as proton pump inhibitors, can alleviate GERD symptoms [1]. Moreover, gastric acid reflux into the esophagus characteristically increases after eating [2], and although GERD patients sometimes complain of nausea and a loss of appetite, the acid reflux into the esophagus that causes GERD may instead be partially due to the amount and content of food. There are various peripheral and central appetite-related hormones involved in the control of appetite and satiation; ghrelin is secreted by the stomach and stimulates appetite and gastrointestinal motility [3], whereas cholecystokinin (CCK) and leptin, which are secreted in response to food intake, suppress appetite [4, 5]. Ghrelin is secreted by X/A-like cells found in the gastric mucosa, and it binds to the growth hormone secretagogue receptor (GHS-R) present at the end of the vagus nerve to stimulate feeding behavior by suppressing the satiety stimulus transmitted by CCK [6, 7]. In contrast, leptin is secreted by fat cells and acts directly on the hypothalamus by crossing the blood-brain barrier, thereby suppressing food intake desire caused by ghrelin [5]. Until date, changes in appetite-related hormones in GERD patients have not been sufficiently characterized. We hypothesized that the progression of GERD may be mediated by the abnormal function of appetite-related hormones. As a first step in elucidating the involvement of appetite-related hormones in GERD, we examined the profiles of peripheral appetite-related peptides, with a focus on changes in ghrelin levels and ghrelin responsiveness.

## 2. Materials and Methods

### 2.1. Animals

Eight-week-old male Wistar rats (CLEA Japan, Tokyo, Japan) were used during the experiment. During testing, 4-5 animals were housed in a single cage and were allowed free access to food and water. Animal rooms were illuminated between 07:00 and 19:00, and temperature and humidity were maintained at constant levels. All tests were performed between 09:00 and 18:00, according to the guidelines of the Experimental Animal Ethics Committee of Tsumura. 

### 2.2. Chemicals

Rat ghrelin was obtained from the Peptide Institute (Osaka, Japan) and was dissolved in 0.9% sterilized physiological saline (Otsuka Pharmaceutical, Tokyo, Japan).

### 2.3. Preparation of GERD Rats

GERD was surgically induced by Omura's method [8]. Rats deprived of food for 24 h were anesthetized with ether. The abdomen was opened using a 2 cm upper-median abdominal incision. The stomach and duodenum were exteriorized, and the boundary between the forestomach and the glandular stomach was sutured with 1-0 silk thread (Natsume Seisakusho, Tokyo, Japan). A precut 2 mm wide 18-Fr Nelaton catheter (Terumo, Tokyo, Japan) was used to cover the area proximal to the pylorus on the duodenal side, and a 5–0 nylon thread (Natsume Seisakusho, Tokyo, Japan) was used to suture and fix it to the surface of the pyloric serous membrane. The stomach and duodenum were placed back into the abdominal cavity, which was then closed. Sham-operated rats were first laparotomized to expose their stomach and duodenum for about 1 min, after which their abdominal cavities were closed. After surgery, rats were fasted for an additional 24 h (resulting in a total of 48 h). GERD-induced animals that were noted to have developed organ adhesions or abscesses or extreme weight loss or weakness were excluded from the experiment. To avoid a dramatic reduction in the sample, more GERD-induced animals were created than sham-operated rats.

### 2.4. Measurement of Body Weight and Food Intake

The rats were housed individually after GERD induction. Daily body weight was measured from the day of surgery, and daily food intake, calculated as the difference between preprandial and postprandial weight of the food, was measured from 2 days after surgery (day 2). On day 9, rats were deprived of food for 24 h and then sacrificed to perform histopathological assessment of the esophagus.

### 2.5. Histopathological Assessment

After 24 h of fasting (day 10), rats were exsanguinated via the abdominal vena cava under ether anesthesia, and the esophagus was excised. Histopathological assessment was performed as previously described [9]. The esophagus was opened with a longitudinal incision and immobilized on a rubber plate with insect pins. The entire esophagus was photographed, and each image was imported into an image analysis software (WinROOF; Mitani Corporation, Tokyo, Japan). Sites showing esophageal mucosal erosion were identified and their total area was measured.

### 2.6. Effect of Exogenous Ghrelin in GERD Rats

During GERD-inducing surgery, rats were anesthetized by intraperitoneal injection of pentobarbital sodium (Kyoritsu Seiyaku, Tokyo, Japan), and a catheter filled with heparin in physiological saline was fixed to the jugular vein. The catheter was passed subcutaneously and pulled out from the back, which was then covered with a metal spring to prevent it from being bitten. After surgery, rats were housed individually and fasted for an additional 24 h. From the next day, rat ghrelin (3 nmol/rat) was administered to the rats once daily through the jugular vein for 6 days. The control group of GERD and sham-operated rats were administered saline. Daily body weight was measured from the day of surgery and daily food intake from day 2 after surgery.

### 2.7. Determination of Plasma Ghrelin and Appetite-Related Hormones

Blood was collected from the abdominal vena cava under ether anesthesia on days 3, 7, and 10 after surgery. Plasma samples were obtained as previously reported [10]. In brief, blood was collected in a tube containing EDTA-2Na (Dojindo Laboratories, Kumamoto, Japan) and aprotinin (Wako Pure Chemical Industries, Osaka, Japan). Blood samples were immediately centrifuged at 4°C, and the supernatant was acidified with 1 mol/L HCl (1/10 volume). Plasma was stored at −80°C until measurement. Plasma ghrelin levels were measured using the Active Ghrelin ELISA Kit and Desacyl Ghrelin ELISA Kit (Mitsubishi Chemical Medience, Tokyo, Japan). Plasma CCK levels were measured with the CCK EIA Kit (Phoenix Pharmaceuticals, Burlingame, CA, USA) using nonacidified plasma samples obtained in the same manner. Plasma leptin levels were measured using the Bio-Plex suspension array system (BioRad Laboratories, Hercules, CA, USA) with the Bio-Plex Pro Rat Diabetes assay panel (BioRad Laboratories).

### 2.8. RNA Extraction, Reverse Transcription, and Real-Time Polymerase Chain Reaction

After collection of blood samples, the stomach and hypothalamus were immediately excised on days 3, 7, and 10 and stored at −80°C until measurement. The tissue was homogenized and total RNA was extracted using the RNeasy Universal Tissue Kit (Qiagen, Valencia, CA, USA). Total RNA from each sample was diluted to 100 ng/*μ*L, allowed to react for 5 min at 70°C, and immediately cooled on ice. An aliquot of 1 *μ*g of total RNA was reverse transcribed using the TaqMan Reverse Transcription Reagents (Applied Biosystems, Foster City, CA, USA) according to the manufacturer's protocol. 

Quantitative polymerase chain reaction (PCR) was performed with the PRISM 7900HT Sequence Detection System (Applied Biosystems) using the TaqMan Universal PCR Master Mix (Applied Biosystems). To compensate for the differences in the amount of total RNA added to each reaction, mRNA expression was normalized to *β*-actin as an endogenous control as expressed by the Δ threshold cycle (Δ*C*_*t*_) value:

(1) $$\begin{array}{c} \Delta C_{t}=2^{\left(-|A\;-\;B|\right)}, \end{array}$$

where *A* is the number of cycles that reached the *β*-actin gene threshold and *B* is the number of cycles that reached the target gene threshold. The set of oligonucleotide primers and fluorescent probes used in TaqMan quantitative PCR was provided by Applied Biosystems: cytoplasmic *β*-actin, Rn00667869_m1; prepro-ghrelin, Rn00572319_m1; GHS-R, Rn00821417_m1; membrane bound *O*-acyltransferase domain containing 4 (ghrelin *O*-acyltransferase; GOAT), Rn02079102_s1; neuropeptide Y (NPY): Rn00561681_m1; agouti-related protein (AgRP): Rn01431703_g1; proopiomelanocortin (POMC): Rn00595020_m1; promelanin-concentrating hormone (MCH): Rn00561766_g1; and hypocretin (prepro-orexin), Rn00565995_m1.

### 2.9. Statistical Analysis

Statistical significance was examined using Student's *t*-test and *P* < 0.05 was considered statistically significant. Data were expressed as the mean ± SEM of each group.

## 3. Results

### 3.1. General Condition and Histology in GERD Rats

**Figure 1:**
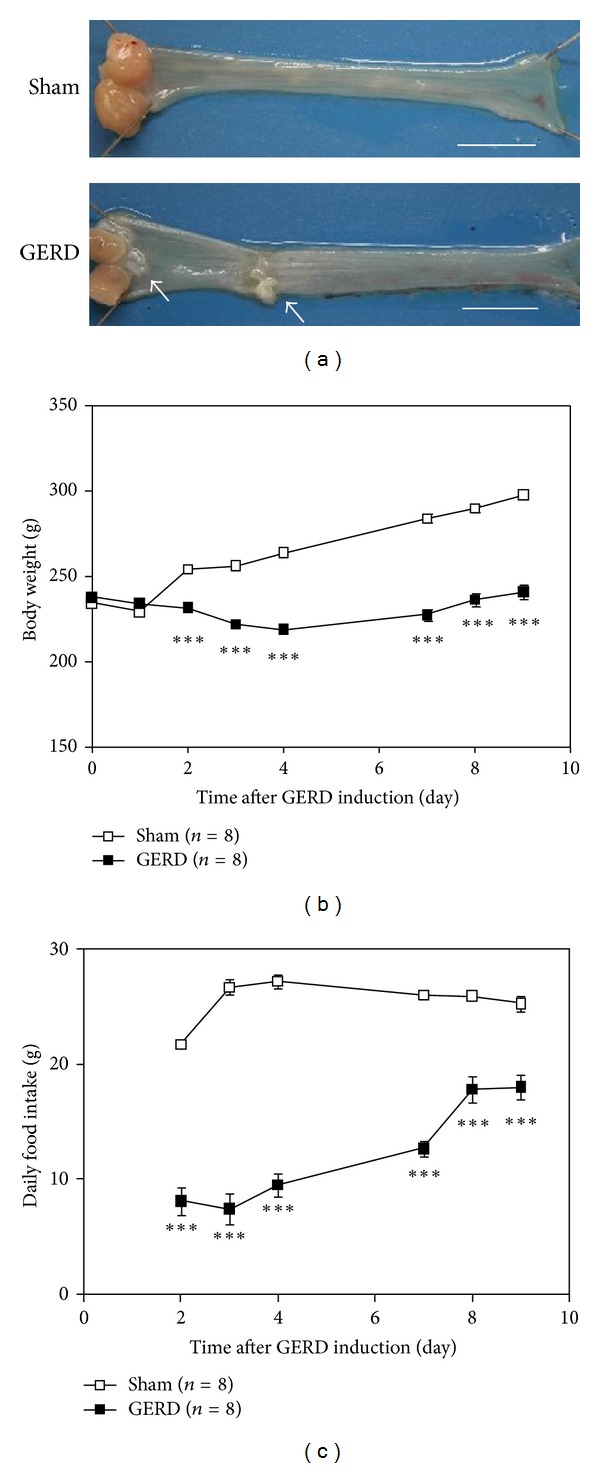
Comparison of esophageal mucosa, body weight, and food intake between sham-operated and GERD rats. (a) Esophagus on day 10 after GERD induction showed obvious erosion.* Bar*, 10 mm. (b) Body weight and (c) daily food intake. ***P < 0.001 versus sham-operated rats.

Mucosal erosion was clearly observed in the esophagus of GERD rats on day 10 (Figure 1(a)). The number of erosion sites in GERD rats was 2.5 ± 0.4 with a total area of 39.7 ± 9.8 mm^2^. Moreover, the body weight and food intake in these rats had significantly decreased compared with those in sham-operated rats (Figures 1(b) and 1(c)).

### 3.2. Changes in Plasma Ghrelin Levels in GERD Rats

**Figure 2:**
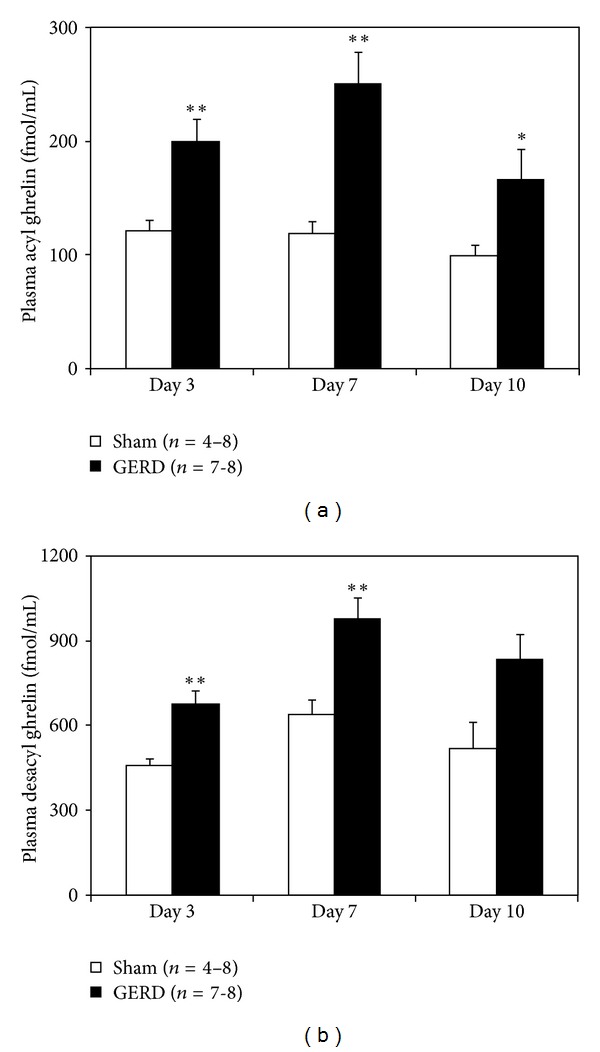
Plasma ghrelin levels in sham-operated and GERD rats on day 3, 7, and 10 after GERD induction. (a) Plasma acyl ghrelin and (b) desacyl ghrelin levels. *, **P < 0.05, 0.01 versus sham-operated rats on each day.

Plasma acyl and desacyl ghrelin levels significantly increased from day 3 to day 10 (Figures 2(a) and 2(b); desacyl ghrelin levels on day 10: sham-operated, 520.4 ± 94.0 versus GERD, 832.9 ± 92.7 fmol/mL; *P* = 0.06).

### 3.3. Plasma Leptin and CCK Levels in GERD Rats

Plasma leptin levels significantly decreased on day 10 (Table 1). There were no significant differences in plasma CCK levels between GERD and sham-operated rats.

### 3.4. Effect of Ghrelin Administration on Body Weight and Food Intake

**Figure 3:**
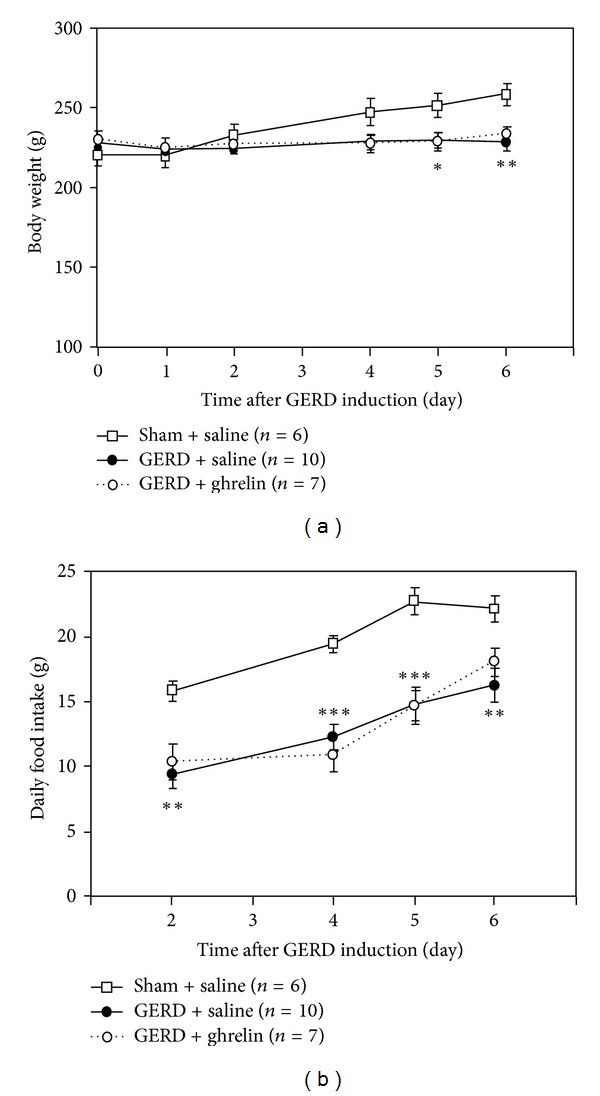
Effect of repeated administration of ghrelin to GERD rats. (a) Body weight and (b) daily food intake in sham-operated and GERD rats. Exogenous ghrelin (3 nmol/rat/day) was intravenously administered to rats once daily. Sham-operated rats and control group of GERD rats were administered saline. There were no significant differences in body weight or food intake between the saline-administered and ghrelin-administered groups in GERD rats. *, **, and ***P < 0.05, 0.01, and 0.001 versus sham-operated rats on each day.

Body weight of GERD rats significantly decreased compared with that of sham-operated rats (Figure 3(a)). The repeated administration of ghrelin to GERD rats had no effect on body weight reduction. Furthermore, there were no differences in daily food intake between GERD rats administered saline and those administered ghrelin (Figure 3(b)). 

### 3.5. Changes in Gastric or Hypothalamic mRNA Expression in GERD Rats

**Figure 4:**
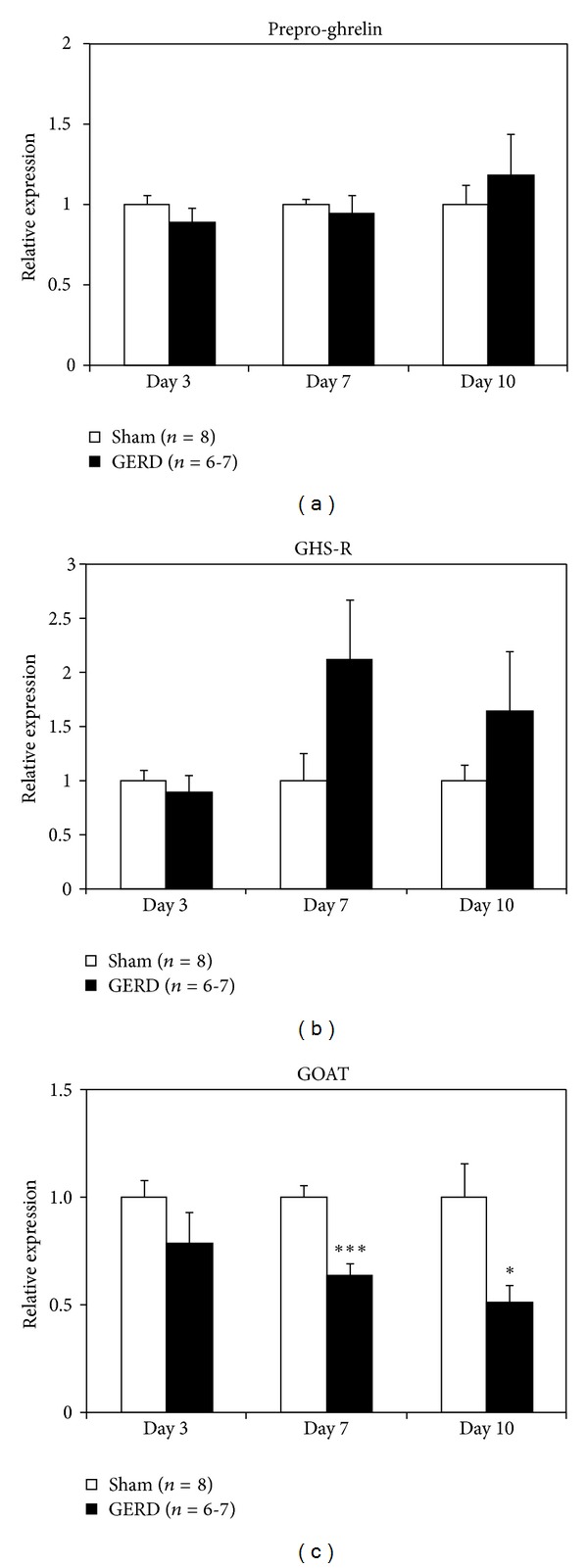
Gastric mRNA expression in sham-operated and GERD rats on days 3, 7, and 10 after GERD induction. (a) Prepro-ghrelin, (b) growth hormone secretagogue receptor (GHS-R), and (c) ghrelin *O*-acyltransferase (GOAT) mRNA expression. *, ***P < 0.05, 0.001 versus sham-operated rats on each day.

**Figure 5:**
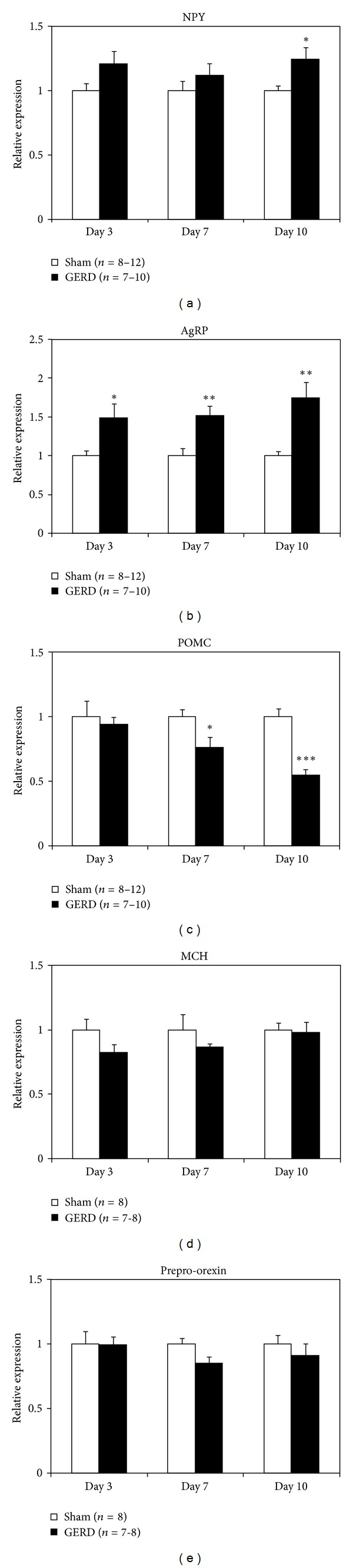
Hypothalamic mRNA expression in sham-operated and GERD rats on days 3, 7, and 10 after GERD induction. (a) Neuropeptide Y (NPY), (b) agouti-related protein (AgRP), (c) proopiomelanocortin (POMC), (d) melanin-concentrating hormone (MCH), and (e) prepro-orexin mRNA expression. *, **, and ***P < 0.05, 0.01, and 0.001 versus sham-operated rats on each day.

There were no major differences in prepro-ghrelin and GHS-R mRNA expression in the stomach throughout the experiment between sham-operated and GERD rats (Figures 4(a) and 4(b)). In contrast, GOAT mRNA expression in GERD rats significantly decreased from day 7 to day 10 (Figure 4(c)). NPY mRNA expression in the hypothalamus of GERD rats significantly increased on day 10 (Figure 5(a)), whereas AgRP mRNA expression significantly increased from day 3 (Figure 5(b)). In contrast, a significant decrease in POMC mRNA expression was observed in GERD rats from day 7 (Figure 5(c)). In addition, MCH (Figure 5(d)) and prepro-orexin (Figure 5(e)) mRNA expression in GERD rats remained unchanged compared with that in sham-operated rats.

## 4. Discussion

Despite the rapid increase in plasma acyl and desacyl ghrelin levels in 24-h fasted GERD rats, their food intake and body weight decreased. Repeated administration of acyl ghrelin did not suppress the reduction in food intake and body weight. Hypothalamic NPY/AgRP neuronal activity, but not MCH or orexin neurons, significantly increased. Peripheral ghrelin signals in GERD rats were sent to the arcuate nucleus in the hypothalamus, but MCH and orexin neurons in the lateral hypothalamic area (LH) might have failed to be activated, leading to inhibition of food intake.

Ghrelin is an orexigenic hormone produced mainly in the stomach [7]. Ghrelin increases food intake and suppresses energy expenditure [3, 11]. In our previous study, we used GERD rats to demonstrate lack of responsiveness through an acute bolus administration of ghrelin (3 nmol/rat) [12]. In comparison with sham-operated rats, the growth hormone secretory effect in GERD rats intravenously administered acyl ghrelin decreased, and acute administration of acyl ghrelin did not suppress the decrease in food intake, gastric emptying, or gastric motility. Repeated administration of acyl ghrelin (3 nmol/rat/day) to GERD rats had no effect on food intake or body weight throughout the experiment in this study. We previously demonstrated that acute intravenous administration of acyl ghrelin at a dose of 3 nmol/rat to sham-operated rats increased food intake significantly [12]. In normal rats, administration of ghrelin at a dose of 1.5 nmol/rat also increased food intake significantly [13]. Food intake did not increase with repeated administration of ghrelin; therefore, GERD rats may require a higher dose of exogenous ghrelin.

The mechanism whereby peripheral blood ghrelin levels are increased in GERD rats is not well understood. There were no differences in relation to prepro-ghrelin or GHS-R mRNA expression in the stomach between sham-operated and GERD rats. These results were not caused by an increase in ghrelin synthesis in the stomach and do not necessarily promote the synthesis of receptors that would increase signal responsiveness. Moreover, expression of the gene encoding GOAT, which is an enzyme that adds an octanoyl group to proghrelin, was significantly decreased. This result is in accordance with the findings that GOAT mRNA may be negatively regulated during long-time fasting [14]. This may be due to negative feedback as a result of abundant acyl ghrelin present in the peripheral blood. Therefore, ghrelin secretion raw the stomach may contribute to the high peripheral blood ghrelin levels.

 GHS-R is a G-protein-coupled receptor, and these receptors typically undergo depolarization after ligand binding [15]. Sustained high plasma ghrelin levels in GERD rats may cause systemic GHS-R depolarization. However, in this study we found that hypothalamic NPY/AgRP mRNA expression significantly increased from day 3 in GERD rats compared with that in sham-operated rats. Ghrelin binds to GHS-R at the end of the vagus nerve in the stomach, stimulates NPY/AgRP neurons present in the hypothalamic arcuate nucleus, and increases NPY/AgRP mRNA expression [3, 13]. Because NPY/AgRP mRNA expression is increased in GERD rats, ghrelin signaling may be maximal. Leptin inhibits NPY/AgRP expression, stimulates POMC neurons, and produces POMC mRNA [16, 17]. Since plasma leptin was only examined on day 10 after surgery, we can only suggest that increased ghrelin levels were inversely correlated with plasma leptin levels. However, in addition to the increased plasma ghrelin levels, decreased plasma leptin levels might have contributed to increased NPY/AgRP and significantly reduced POMC mRNA expression in GERD rats.

In this study, prepro-orexin and MCH mRNA expression was not altered in GERD rats. The orexigenic signal, via the activation of the NPY/AgRP neurons, is transmitted to the LH, leading to the activation of orexin or MCH neurons [18–21]. Orexin and MCH are primarily synthesized in the LH when these neurons are activated. MCH-1R antagonism or depletion of the peptide results in hypophagia, and MCH-1R-deficient mice are lean [22–24]. It is well known that a reward system, including appetite or learning, is mediated by activation of these neurons via the dopamine neurons [25, 26] in the ventral tegmental area or nucleus accumbens. We speculated that orexin or MCH neuron activation might be suppressed in GERD rats, leading to the inhibition of food intake. However, a detailed mechanism of action and the identity of the factors that cause suppression of these neurons is still unknown. Further study is needed to clarify why orexin or MCH neurons are unresponsive.

Currently, there exists no GERD model that suitably reflects human GERD. The model presented in the current experiment describes an extensive operation involving ligation of the forestomach and fixing of a ring to the pyloric region, making it differ greatly from human GERD. Furthermore, although these are preliminary results, administration of an effective dose of PPI [27] did not affect initial body weight, food intake, or ghrelin concentration in GERD rats. It is possible to conclude that overexposure of the esophagus to stomach acid is not involved in the increased secretion of ghrelin or abnormal food intake-related factor expression. Moreover, because oral administration of Cisapride clearly improved gastric emptying in the present model [12], it appears that treatment with a pyloric region ring and ligation does not cause an irreversible reduction in gastric motility. However, the possibility that extensive surgery affects food intake-related parameters could not be excluded. The validity of the GERD model used in this study needs to be sufficiently verified in the future. In addition, the effect of physical impairment and stomach acid exposure in surgery on food intake-related parameters and hormone levels needs to be carefully examined.

## 5. Conclusion

In comparison to normal rats, GERD rats characteristically have increased peripheral acyl ghrelin levels, decreased leptin levels, and might have impaired ghrelin signal transmission. However, it remains necessary to verify the validity of the model used and to further examine details regarding PPI administration. 

## Figures and Tables

**Table 1:** Plasma leptin and cholecystokinin (CCK) levels in sham-operated and GERD rats on day 10 after GERD induction.

	Sham (*n* = 4 or 8)	GERD (*n* = 8)
	(pg/mL)	(pg/mL)
Leptin	714.6 ± 49.3	358.1 ± 78.5*
CCK	133.8 ± 8.2	112.3 ± 6.8

*P < 0.05 versus sham-operated rats.
